# Contrast induced nephropathy; recent findings

**Published:** 2013-07-01

**Authors:** Saeed Mardani, Parto Nasri, Maryam Tavakoli

**Affiliations:** ^1^Department of Internal Medicine, Shahrekord University of Medical Sciences, Shahrekord, Iran; ^2^School of Medicine, Isfahan University of Medical Sciences, Isfahan, Iran; ^3^Department of Internal Medicine, Isfahan University of Medical Sciences, Isfahan, Iran

**Keywords:** Contrast nephropathy, Renal failure, Renoprotection

## Abstract

Contrast induced nephropathy is one cause of acute renal failure. Contrast induced nephropathy is the third most common cause of hospital-acquired acute renal failure. The incidence of contrast induced nephropathy in the general population is 0.6% to 2.3%, but when focusing on specific high-risk patients the incidence can increase to more than 40%. Studies suggest that intravenous hydration is the most effective strategy to prevent contrast induced nephropathy. Hydration is inexpensive and is usually risk-free. Administration of optimal fluids before and after the contrast procedure allows for increased urine output and improved outcomes.

**Implication for health policy/practice/research/medical education:**

Contrast induced nephropathy is one cause of acute renal failure. Contrast induced nephropathy is the third most common cause of hospital-acquired acute renal failure. The incidence of contrast induced nephropathy in the general population is 0.6% to 2.3%, but when focusing on specific high-risk patients the incidence can increase to more than 40%. Studies suggest that intravenous hydration is the most effective strategy to prevent contrast induced nephropathy. Hydration is inexpensive and is usually risk-free. The goal of prevention is to protect the renal tubules from prolonged contact with contrast material, because permanent damage can occur at the time of contact. Administration of optimal fluids before and after the contrast procedure allows for increased urine output and improved outcomes.

## Introduction

Contrast induced nephropathy is one cause of acute kidney failure. Contrast induced nephropathy is the third most common cause of hospital-acquired acute renal failure (1). The incidence of contrast induced nephropathy in the general population is 0.6% to 2.3%, but when focusing on specific high-risk patients the incidence can increase to more than 40% (1,2).

Contrast induced nephropathy is associated with an increased risk of prolonged hospital stay, increased risk of nosocomial complications, potential need for dialysis, increased health care costs and mortality (1-3).

Identification of patients potentially susceptible of developing contrast induced nephropathy before their exposure is important, since modification of the ways we administer contrast medium can lead to a decrease in acute kidney injury (1-4).

Diagnosis of contrast induced nephropathy is most often based on an increase in the serum level of creatinine after exposure to a contrast agent.

## Diagnostic criteria for contrast-induced nephropathy

1. Exposure to contrast agent.
2. Increase in serum level of creatinine of 0.5 mg/dl or 25% greater than baseline.
3. Increase in serum level of creatinine occurs 48-72 hours after administration of contrast agent and persists for 2-5 days.
4. Alternative major injuries are ruled out.

## Risk factors

Addressing and resolving modifiable risk factors before administration of contrast agents decrease a patient’s risk of contrast induced nephropathy (2-5).

Risk factors for development of contrast-induced nephropathy are; Dehydration, hyperosmolar media, administration of >100 mL of contrast media, recent administration of contrast media, hypotension, use of nephrotoxic agents, anemia, shock, sepsis, use of intra-aortic balloon pump, preexisting kidney impairment, diabetes, ventricular dysfunction: ejection fraction <40%, hypercholesterolemia and age >75 years (3-6).

Patients with existing impaired renal function or diabetes mellitus (type 1 and type 2) are at the highest risk.

Diabetes mellitus putatively predisposes host kidneys to ischemic injury (from macro- or micro-vascular stenosis), increases oxidative stress and free radical damage, as well as endothelial dysfunction. In addition to the impact of a baseline diabetes mellitus, pre-procedural glucose level higher than 200 mg/dl, is also a risk factor for contrast-induced nephropathy (2-7).

Advanced age is another risk factor that enhances the probability of developing contrast-induced nephropathy. Age higher than 75 can associate with a 1.5-5 fold elevated risk, while every one-year increment carries a 2% increased risk.

The physiologic degeneration of the kidney which named as aging kidney, both structurally and functionally, and the ability of recovery after various nephrotoxic insults also dampens in this population (5-9).

One-third of patients with serum creatinine level higher than 2.0 mg/dl receiving contrast media for radiographic studies will develop contrast-induced nephropathy (1-7).

Hemodynamic instability and anemia are as a factor that reduces tissue oxygenation and predisposes to contrast-induced nephropathy (2-7).

The most common procedures associated with contrast-induced nephropathy are coronary angiography and contrast-enhanced computed tomography. Patients who undergo coronary angiography are at highest risk for contrast-induced nephropathy. Often the procedure is done under emergent conditions, and patients may be volume depleted, and their hemodynamic status may be less than optimal (3-9).

Congestive heart failure (grade 3-4) is associated with elevated risk of contrast-induced nephropathy use of angiotensin-converting-enzyme inhibitors or angiotensin receptor blockers is associated with 2.5-3.0 fold higher risk of developing contrast-induced nephropathy after coronary angiography. On the contrary, withdrawal of angiotensin-converting-enzyme inhibitor or angiotensin receptor blocker before coronary procedures does not seem to reduce the risk of contrast-induced nephropathy (1-5).

Other nephrotoxic substances including; nonsteroidal anti-inflammatory drugs, calcineurin inhibitors, loop diuretics, aminoglycosides, amphotericin B, vancomycin, chemotherapeutic agents and metformin (6-9).

Even small volumes of contrast media (~30 ml) might trigger kidney injury in high-risk patients. For every 100 ml increase in the amount of contrast media used, there is a concomitant 12% increase of the risk. Adjustment of the contrast volume to one’s body weight and serum creatinine level could minimize the risk (8,9).

Intra-arterial injection of contrast media carries a higher risk of contrast-induced nephropathy than intravenous use. However, no mechanisms have been provided to explain this phenomenon. Some reasons are as the dose used in intravenous enhancement for computed tomography (CT) is usually lower than that for arteriography; patients who received contrast-enhanced CT are usually less hemodynamically unstable than ones receiving intra-arterial (2-9).

## Pathogenesis

The pathogenesis of contrast-induced nephropathy is not clear. Studies suggest that contrast-induced nephropathy, is due to a combination of toxic and ischemic injury to the kidney tubular cells. Proximal and distal tubular injury occurs at the moment of contact with contrast media and is thought to be due to an interplay of intrarenal vasoconstriction, medullary hypoxia, and direct tubular cell death (3-9).

Mechanisms underlying contrast-induced nephropathy, include direct cytotoxic effects, auto-, and paracrine factors that perturb kidney hemodynamics, altered rheological properties that affect kidney hemodynamics and tubulodynamics, and regional hypoxia (2-8).

Contrast media intravascular injection can increase the activity of a variety of vasoactive substances, including vasopressin, angiotensin II, dopamine-1, endothelin and adenosine, while decrease the activity of renal vasodilators such as nitric oxide and prostaglandins (3-10).

Patients with diabetes and kidney failure have an increased risk for contrast-induced nephropathy, because of a reduction in endogenous vasodilators such as nitric oxide and prostaglandins, which results in a decrease in kidney blood flow and glomerular filtration rate (2-9).

Contrast agents may trigger the release of endothelin and adenosine from endothelial cells, increasing vasoconstriction, and decrease the release of prostaglandins, preventing vasodilatation, hence, decreasing oxygen in the outer medulla. Impairment in flow leads to hypoxia and decreased nutrient delivery to tubular epithelial cells, resulting in increases in reactive oxygen species, causing breakdown of the epithelial cyto-structure with loss of cell parity and death of the cell (3-8).

Length of exposure to a contrast agent is related to the extent of damage of kidney tubular cells. Contrast agents are thought to produce prolonged vasoconstriction of the arteriole and stasis of contrast material in the renal vasculature, resulting in medullary ischemic injury and death of proximal and distal kidney tubular cells (1-5).

The osmolality of contrast media may play a role in the pathogenesis of contrast-induced nephropathy. A relationship exists between osmolality and viscosity, thereby increasing the resistance to flow in kidney tubules. High osmolal agents diminish the deformability of erythrocytes, thereby increasing the cells’ stiffness and making the flow of red blood cells through the capillaries more difficult (8-12).

Hypovolemia triggers physiological countermeasures aiming at volume preservation, especially, activation of the renin-angiotensin system and of vasopressin. Angiotensin II and vasopressin augment tubular fluid resorption, which diminishes urine flow rate. In addition, angiotensin II elicits kidney vasoconstriction, which aggravates contrast media induced medullary hypoperfusion (4-10).

The tubular toxicity of contrast medium can be demonstrated in the pathological changes it induces, consisting epithelial vacuolization, apoptosis, cellular necrosis and interstitial inflammation. Contrast media can additionally reduce antioxidant enzyme activity within the kidney, and free radical mediated cytotoxicity of the renal tubular cells has been detected in these models (2-8).

## Prevention

Patients with hypertension, congestive heart failure, diabetes mellitus or potentially changing renal function should receive a baseline renal function testing, and if possible, a nephrology consultation could be obtained (3-7).

Hyperglycemic status should be properly managed before administration of contrast media. Agents such as nonsteroidal anti-inflammatory drugs, diuretics, and possibly angiotensin-converting-enzyme inhibitors should be discontinued 1-2 days before administration of contrast media (1-7).

The amount of contrast media volume should be as little as possible, and the choice of contrast media should be iso-osmolar or low osmolar agents (1-5).

This cytotoxic effect does not seem to be caused by iodine or osmolality lesser than 830 mosm/L (5-9).

The goal of prevention is to protect the renal tubules from prolonged contact with contrast material, because permanent damage can occur at the time of contact (2-8).

Repeated exposure should be delayed for 48 hours in patients at-risk of developing contrast-induced nephropathy (1-6).

Studies suggest that intravenous hydration is the most effective strategy to prevent contrast-induced nephropathy. Hydration is inexpensive and is usually risk-free. Administration of optimal fluids before and after the contrast procedure allows for increased urine output and improved outcomes (1-8).

The benefit of adequate volume expansion includes improving renal blood flow, inducing diuresis with dilution of contrast media within kidney tubules, suppression of the renin-angiotensin-aldosterone system, and less reductions in the kidney production of endogenous vasodilators (3-9).

Intravenous hydration is more favorable than oral hydration. The most effective solution for preventing of contrast-induced nephropathy is isotonic saline (0.9%).

No benefit from forced diuresis with intravenous crystalloid, furosemide, mannitol or low dosed dopamine therapy, compared with hydration alone in at-risk patients. Isotonic hydration is superior to half-isotonic hydration in the efficacy for prevention of contrast-induced nephropathy. It is recommended now that intravenous hydration should start 12 hours before and continue for 12 hours after (2-9).

Two strategies are available to prevent an acidic environment and formation of free radicals in the renal tubules: sodium bicarbonate and N-acetylcysteine. N-acetylcysteine increases production of nitric oxide, which has vasodilatory capabilities, and the concentration of glutathione, which acts as a free radical scavenger. Compared with infusion of normal saline alone, administration of N-acetylcysteine in conjunction with infusions of normal saline significantly decreased the risk for contrast-induced nephropathy (3-11).

The most common protocol of N-acetylcysteine is to give this agent orally 600 mg twice a day for 24 hours on the day before and the day of procedure (3-11).

Sodium bicarbonate may result in urine alkalinization and reduce the generation of free radical through scavenging reactive oxygen species (3-7).

Fenoldopam mesylate is a selective dopamine-1 receptor agonist that produces systemic and kidney artery vasodilatation. It is found to exhibit desirable renal effects including decrease in renal vascular resistance and increase in renal blood flow, glomerular filtration rate, with natriuresis. The routine use of fenoldopam to protect against contrast-induced nephropathy could not be recommended (2-7). Other agents such as ascorbic acid, theophylline, statin can be used to protect of contrast-induced nephropathy but these agents routinely not recommended.

## Conclusion

Contrast induced nephropathy is one cause of acute renal failure. Contrast induced nephropathy is the third most common cause of hospital-acquired acute renal failure. The incidence of contrast induced nephropathy in the general population is 0.6% to 2.3%, but when focusing on specific high-risk patients the incidence can increase to more than 40%. Studies suggest that intravenous hydration is the most effective strategy to prevent contrast induced nephropathy. Hydration is inexpensive and is usually risk-free. The goal of prevention is to protect the renal tubules from prolonged contact with contrast material, because permanent damage can occur at the time of contact. Administration of optimal fluids before and after the contrast procedure allows for increased urine output and improved outcomes.

## Authors’ contributions

All authors wrote the paper equally.

## Conflict of interests

The authors declared no competing interests.

## Ethical considerations

Ethical issues (including plagiarism, data fabrication, double publication) have been completely observed by the authors.

## Funding/Support

None.
